# Active Isoflavones of Osaje Orange Fruits against *Staphylococcus aureus*

**DOI:** 10.1002/cbdv.202502220

**Published:** 2025-11-08

**Authors:** Gabin Thierry M. Bitchagno, Debbie Mulligan, Paula Coates, Erin Garcia, Sohini Bhatia, Scott Bintrim, Monique S. J. Simmonds

**Affiliations:** ^1^ Royal Botanic Gardens, Kew Richmond UK; ^2^ The Procter & Gamble Company, Mason Business Center Mason Ohio USA; ^3^ The Procter & Gamble Company Reading UK

**Keywords:** isoflavone, *Maclura pomifera*, metabolomic, Moraceae, *Staphylococcus aureus*, structure elucidation

## Abstract

The genus *Maclura* is known for producing prenylated isoflavones with various biological activities, including antibacterial properties. Of the multiple isoflavones reported from some of its species, like *Maclura pomifera* (Rafin.) Schneider, the main active principle, remains unknown. A bio‐guided analysis of the EtOAc fruit extract of *M. pomifera* resulted in the isolation of the active ingredient against *Staphylococcus aureus*. Examination of the most active fractions also led to a novel isoflavone, dihydroxyosajin (**1**), whose structural features were determined using spectroscopic and spectrometric methods. The proportions of the key active compounds in the plant material were evaluated using a multiple reaction monitoring method. Pomiferin and osajin were identified as the two predominant compounds. These compounds were present in greater amounts than scandenone and auriculasin, which are often reported as the major constituents in the species. The study recommends verifying that the acquired samples contain the target compounds in effective proportions.

## Graphical abstract

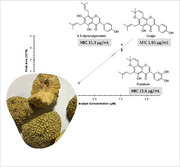

## 1 Introduction

*Maclura pomifera* (Rafin.) Schneider, also known as osaje orange, horse apple, hedge apple, hedge ball, or bois d'arc, was recorded as being native to the southeastern coast of the US and was further introduced across other US states and countries around the World, including Argentina, southern Europe, and the UK. The plant has been the subject of multiple studies on the structural variety of compounds it contains and the variety of biological applications associated with the species [1, 2]. From a chemical standpoint, the plant produces mostly prenylated flavonoids [3] of which osajin and pomiferin are the most studied [4, 5]. These are compounds consisting of a genistein or orobol unit, to which prenyl and dimethylpyrene groups are grafted onto the A‐ring 6 and 8 positions, respectively. Together with scandenone and auriculasin, these compounds are reportedly the most abundant components of the plant aerial parts, including the fruits [6, 7].

Pomiferin and osajin have been reported to have antibacterial, antidiabetic, anticancer, anti‐inflammatory, and antioxidant activities during in vitro and in vivo experiments [7]. These activities have almost always been associated with isoflavones. Especially the prenylated or geranylated flavonoids that are reported to confer resistance of the plant to pathogens, particularly bacteria. However, this resistance was only to Gram‐positive bacteria. The activity of the prenylated analogues was associated with their lipophilicity [8]. In this respect, reports have associated *Maclura*’s isoflavones with significant potency against Gram‐positive bacteria such as *Staphylococcus aureus* [7]. Of the multiple (>10) isoflavones reported from *M. pomifera*, the main active principle remains unknown. The present report uses bio‐guided analysis to identify the active compounds.

## 2 Results and Discussion

**FIGURE 1:**
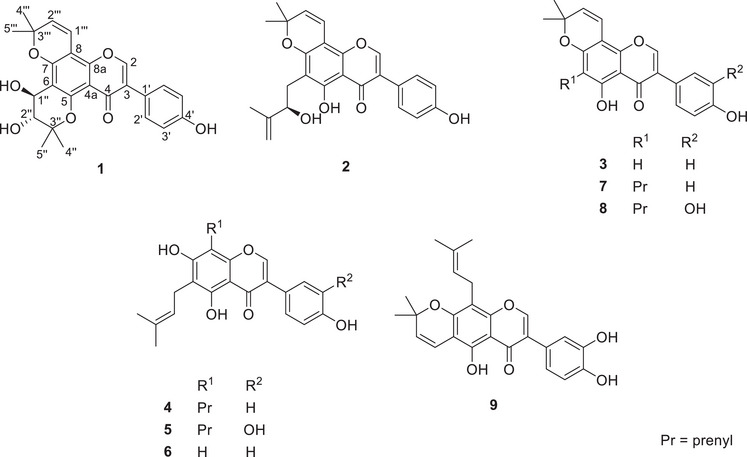
Structures of isolated compounds from *M. pomifera* fruits. Euchrenone b9 (**2**), derrone (**3**), 6,8‐diprenylgenistein (**4**) and 6,8‐diprenylorobol (**5**), 6‐prenylgenistein (**6**), osajin (**7**), pomiferin (**8**), and auriculasin (**9**).

The EtOAc and hexane extracts were the most active against *S. aureus* with minimum inhibitory concentration (MIC) values of 3.9 and 7.8 *µ*g/mL, respectively. None of the extracts showed activity against the other microbes. The liquid chromatography‐mass spectrometry (LC‐MS) and ^1^H nuclear magnetic resonance (NMR) profiles of both the EtOAc and hexane extracts were superimposable. Subsequently, the EtOAc extract alone was considered for further analysis and, based on the profile of compounds, was split into four fractions (E1–E4) using semi‐automatic flash chromatography. The fractions were again tested against *S. aureus*, and E2 and E3 exhibited similar levels of potency as the extract, with a MIC of 15.6 and 31.3 *µ*g/mL, respectively. Consequently, an untargeted analysis of fractions E2 and E3 was undertaken and resulted in the isolation of nine (**1**–**9**) prenylated flavonoids, including the two major constituents (**7** and **8**) of the plant. Compound **7** was the main component in fraction E2, whereas compound **8** was the main component in fraction E3. Both compounds were the main constituents of fraction E4. In addition, an oxidized isoflavone (**1**) was isolated with the structure and occurrence described herein for the first time (Figure 1). The structures of all isolates were determined by means of NMR and MS facilities.

Compound **1** was a yellowish powder. Its negative‐mode HRESIMS exhibited a protonated molecular ion peak [M + H]^+^ at *m/z* 437.1592 for the molecular formula C_25_H_25_O_7_^+^ (calcd for C_25_H_25_O_7_^+^, *m/z* 437.1595). Its ^1^H and 2D NMR spectra (Figures S3‐S6) evidenced signals at *δ*_H_ 7.29 (d, *J* = 8.7 Hz, H‐2'/H‐6') / *δ*_C_ 130.5 (C‐2'/C‐6') and 6.83 (d, *J* = 8.7 Hz, H‐3'/H‐5') / *δ*_C_ 115.2 (C‐3'/C‐5') of a *para*‐substituted aromatic system as judged by HMBC interactions (Figure S6) from H‐2'/H‐6' to quaternary carbons at *δ*_C_ 126.5 (C‐3) and 157.3 (C‐4') and from H‐3'/H‐5' to carbon C‐1' (*δ*_C_ 123.5) (Table 1). The spectrum further exhibited an olefinic singlet at *δ*_H_ 7.78 (s, H‐2) with HMBC cross peaks to a carbonyl C‐4 (*δ*_C_ 176.8), a second oxygenated aromatic carbon C‐8a (*δ*_C_ 154.3), and C‐1'. Interestingly, the spectra evidenced two additional pairs of doublets at *δ*_H_ 6.72 (d, *J* = 10.2 Hz, H‐1''') and 6.72 (d, *J* = 10.2 Hz, H‐2'''), then at *δ*_H_ 4.72 (d, *J* = 6.4 Hz, H‐1'') and 3.72 (d, *J* = 6.4 Hz, H‐2''). The first set linked up with two geminal methyl singlets at *δ*_H_ 1.54 (s, H‐5''') and 1.49 (s, H‐4''') into a 2,2‐dimethyl‐2*H*‐chromene as supported by HMBC interactions from H‐4'''/H‐5''' to C‐3''' (*δ*_C_ 79.5) and C‐2''' (*δ*_C_ 127.2); from H‐1''' to C‐8a and C‐3''' and from H‐2''' to C‐8 (*δ*_C_ 100.6). The second pair of doublets was also associated with another couple of methyl singlets at *δ*_H_ 1.51 (s, H‐5'') and 1.28 (s, H‐4'') into a 2,2‐dimethylchromane‐3,4‐diol as judged by HMBC interactions from both methyl groups to C‐3'' (*δ*_C_ 80.5) and C‐2'' (*δ*_C_ 73.9); from H‐1'' to C‐5 (*δ*_C_ 154.9) and C‐3'' and from H‐2'' to C‐6 (*δ*_C_ 109.6). The coupling constant between H‐1'' and H‐2'' in compound **1** indicates a *trans*‐diaxial configuration of the chromane as compared to a *cis*‐configured ring in macluraflavone L, the orobol version of compound **1** [9]. Thus, compound **1** was characterized as a genistein analogue with both chromene and chromane groups fused to ring‐A of the core structure and was proposed the trivial name dihydroxyosajin.

**TABLE 1:** ^1^H (400 MHz) and ^13^C (100 MHz) nuclear magnetic resonance (NMR) spectroscopic data for compound **1** (*δ* in ppm).

Position	1	
1		
2	7.78, s	150.2, CH
3		126.5, C
4		176.8, C
4a		95‐110, C
5		154.9, C
6		109.6, C
7		156.2, C
8		100.6, C
8a		154.3, C
1'		123.5, C
2'	7.29, d (8.7)	130.5, CH
3'	6.83, d (8.7)	115.2, CH
4'		157.3, C
5'	6.83, d (8.7)	115.2, CH
6'	7.29, d (8.7)	130.5, CH
1''	4.72, d (6.4)	67.0, CH
2''	3.72, d (6.4)	73.9, CH
3''		80.5, C
4''	1.28, s	19.6, CH_3_
5''	1.51, s	25.1, CH_3_
1'''	6.72, d (10.2)	114.9, CH
2'''	5.60, d (10.2)	127.2, CH
3'''		79.5, C
4'''	1.49, s	28.0, CH_3_
5'''	1.54, s	28.1, CH_3_

The ^13^C NMR values are retrieved from the HSQC and HMBC spectra, as the ^13^C NMR spectrum could not be recorded during analysis.

Further isoflavones (**2**–**9**) were isolated as previously reported compounds following confirmation of their structures by means of 2D NMR and LC‐MS/MS analysis. Euchrenone b9 (**2**) [10], derrone (**3**) [11], 6,8‐diprenylgenistein (**4**) [12] and 6,8‐diprenylorobol (**5**) [13], are reported herein for the first time in *M. pomifera* while 6‐prenylgenistein (**6**) [3], osajin (**7**) [14], pomiferin (**8**) [14] and auriculasin (**9**) [15] were previously reported from *M. pomifera*. It is worth noting the absence of scandenone, which in other studies has been reported to occur in high yield in *Maclura*’s fruits [16]. Compounds **4** and **6**–**9** were isolated in amounts that enable them to be tested in the bacterial assays. None of the compounds was active against either *Escherichia coli* or *A. brasilliensis*. However, all compounds exhibited activity against *S. aureus* with MICs ranging from 1.95 *µ*g/mL to 31.3 *µ*g/mL (Table 2). Compound **7** (MIC 1.95 *µ*g/mL or 4.8 *µ*M) was the most active sample, followed successively by compounds **6** (MIC 7.8 *µ*g/mL or 23.5 *µ*M), **8** (MIC 15.6 *µ*g/mL or 37.1 *µ*M), **4** (MIC 31.3 *µ*g/mL or 77.3 *µ*M), and **9** (MIC 31.3 *µ*g/mL or 74.5 *µ*M). In addition, compound **7** and the hexane extract were bacteriostatic, while the others were bacteriocidal. The level of activity observed in the study aligns well with reports available in the literature where osajin was reported most active than scandenone and 6,8‐diprenylgenistein [17].

**TABLE 2:** Results of the antibacterial activity (*µ*g/mL) of extracts, fractions (E1–E4), and isolated compounds **4**, **6**–**9** against *Staphylococcus aureus*.

	Extracts			Fractions				Isolated compounds					Reference
	**Hex**	**EtOAc**	**60%MeOH**	**E1**	**E2**	**E3**	**E4**	**4**	**6**	**7**	**8**	**9**	**Piroctone olamine**
MIC	7.8	3.9	500	>1000	15.6	31.3	15.6	31.3	7.8	1.95	15.6	31.3	15.6
MBC	62.5	3.9	>1000	>1000	62.5	62.5	62.5	125	15.6	62.5	62.5	125	250
R	8	1	—	—	4	2	4	4	2	32	4	4	16

R = MBC/MIC; Hex, EtOAc, and 60% MeOH = Serial extracts in hexane, ethyl acetate, and 60% MeOH in water; E1–E4 = fractions collected from the flash chromatography; **4** = 6,8‐diprenylgenistein; **6** = 6‐diprenylgenistein; **7** = osajin; **8** = pomiferin; **9** = auriculasin.

During fractionation, the primary compounds **7** and **8** diffused across fractions E4, making it impossible to accurately quantify them based on the proportions obtained after isolation and purification. Therefore, it was necessary to return to the original extract and assess their actual quantities within the plant material. To achieve this, a new EtOAc extract was prepared directly from the powdered dried fruits of the plant, aiming to concentrate the compounds previously separated into the hexane and EtOAc extracts into a single extract. The most abundant compounds, **4**, **7**, and **8**, were quantified using the multiple reaction monitoring (MRM) method. The MS2 spectra for each compound were analyzed at a normalized energy level of 10 to identify the optimal transition for each compound. Five different concentrations per compound were prepared in triplicate and analyzed through the MRM setup, with data processed using Skyline software [18]. Its package allows for the extraction of peak areas from both precursor and product ions, which then serve to draw a calibration curve for quantification. As a result, 6,8‐diprenylgenistein (**4**), osajin (**7**), and pomiferin (**8**) accounted for 1.5%, 2.23% and 2.38% of the dried material, respectively (Figures S7‐S9). The concentrations of the dried material in osajin and pomiferin are consistent with previously reported data [7]. Auriculasin, often cited as a major plant component, was present in amounts significantly lower than its analogue pomiferin, while scandenone was not detected.

## 3 Conclusions

Overall, the results support the fact that isoflavones in *Maclura pomifera* are associated with the antibacterial activity of this species. Osajin and pomiferin were the main compounds and showed the most activity against *S. aureus* along with 6‐diprenylgenistein. Considering its yield and bioassay potency (MIC = 4.8 *µ*M), osajin should be considered the main active principle in fruits. The difference in the proportion of the major constituents of the plant compared to those reported in the literature is an opportunity to highlight significant differences in *Maclura* samples that may be found on the market or from suppliers. It is therefore recommended that researchers ensure that the samples they acquire contain compounds of interest.

## 4 Experimental

### 4.1 Essential Experimental Procedures/Data

LC–MS grade solvents (acetonitrile, methanol) and formic acid were obtained from Fisher Scientific (Loughborough, UK), and milliQ water was used for HPLC and LC‐MS analysis. NMR spectra were acquired on a Bruker Avance‐III (^1^H NMR: 400 MHz and ^13^C NMR: 100.1 MHz) spectrometer equipped with a 5 mm cryoprobe. Chemical shifts were referenced to residual solvent signals and reported in parts per million (ppm). Spectra were processed using Bruker NMR academic Topspin software. Mass spectra were collected on a Orbitrap Exploris mass spectrometer, equipped with a Vanquish diode array detector (VH‐D10) coupled to an Orbitrap Exploris 120 with a heated ESI source (Thermo Scientific, Germany), acquired in both negative and positive modes with a resolution of 60 000 over *m/z* 125–1800 under various acquisition parameters like source voltages, sheath gas, auxiliary gas, sweep gas and capillary temperature set to 2.5 kV (negative mode) and 3.5 kV (positive mode), 50 (arbitrary units), 10 (arbitrary units), 1 (arbitrary units) and 350°C, respectively. Automatic MS–MS fragmentation was performed on the top four ions of the TIC using an isolation width of *m/z* 2. High‐energy C‐trap dissociation with a normalized collision energy of 40 and an activation time of 0.1 ms was used to fragment ions. Collected data were inspected using Xcalibur v. 4.2.47 (Thermo Fisher Scientific). Chemical profiling of extracts was conducted on a Biotage Isolera One system for splitting extracts into small fractions and a Waters Alliance 2695 HPLC system for isolation of compounds. A reversed‐phase Discovery HS C‐18 column (5 µm, 10 mm × 250 mm i.d., Supelco, UK) maintained at 35°C served in compound isolation and purification over a gradient of acetonitrile+0.1% formic acid (A) and water (B).

### 4.2 Plant Material

The fruits of *Maclura pomifera* were collected from the living collection at RBG Kew, accession No 1910–66601, in November 2021. The plant tissues were freeze‐dried, milled into fine powders, and kept in the dark before being used.

### 4.3 Extraction and Purification of Compounds

Part of the milled plant materials (70 g) was serially extracted in solvents with increasing polarities starting with *n*‐hexane, then EtOAc, and 60% MeOH, affording dried extracts of 2.46, 4.53, and 5.0 g, respectively. The EtOAc extract was split into four small fractions (E1–E4) on the Biotage Isolera system using a stepwise gradient of EtOAc in hexane starting from 100% hexane to 100% EtOAc, 10% increment, and 3 column‐volumes (CV) at each floor. The fractions were dissolved in CDCl_3_ and submitted to ^1^H NMR, then to 2D NMR for chemical profiling. As rightly put above, only fractions E2 (826 mg) and E3 (1.29 g) were further prepared for compound isolation. Fraction E2 was dissolved in 4 mL of acetonitrile (ACN) + 10% dimethyl sulfoxide (DMSO) and injected into the Waters system, eluting with a constant flow rate of 2 mL/min of a linear gradient of acetonitrile (B) in water (A) (0–5 min, 35% B; 5–55 min, 35%–55% B, 55–65 min, 100% B and 65–75 min, 35% B). Compounds were detected at 210, 254, 300, and 354 nm and collected by time into glass tubes. Cumulative fractions from forty injections of 50 *µ*L each were collected and dried using a GeneVac concentrator (Genevac, Suffolk, UK). Likewise, fraction E3 was eluted with a linear gradient of acetonitrile (B) in water (A) (0–5 min, 35% B; 5–55 min, 35%–55% B, 55–65 min, 100% B, and 65–75 min, 35% B). Compounds were detected, collected, and dried under the same conditions. Compounds **1** (1.2 mg), **2** (1.2 mg), **3** (0.8 mg), **4** (2.9 mg), **5** (0.7 mg), **7** (128.7 mg), **8** (3.0 mg), and **9** (1.8 mg) were collected from E2 while fractions E3 led to compounds **6** (2.9 mg), **7** (2.3 mg), **8** (181.3 mg) and **9** (4.8 mg).

### 4.4 Dihydroxyosajin (**1**)

Yellowish powder; UV λ_max_ 218, 266 nm; ^1^H and ^13^C NMR (MeOH‐*d*_4_), see Table 1; (+)‐HRESIMS *m/z* 437.1592 [M + H]^+^ (calcd for C_25_H_25_O_7_^+^, *m/z* 437.1595).

### 4.5 Quantification Method

Ultra‐performance LC‐MS (UPLC–MS) analyses were performed using a Vanquish UHPLC system (Thermo Scientific, Waltham, MA, USA) coupled with a 100 Hz photodiode array (PDA) detector and an Orbitrap Exploris 120 Mass Spectrometer (Thermo Scientific, Waltham, MA, USA). For mass spectrometric analysis, electrospray ionization‐tandem MS (ESI‐MS/MS) was conducted in positive ionization mode, utilizing nitrogen as the drying and nebulizing gas. The gas flow rate was set to 50.0 L/min at 350°C, with a nebulizer pressure of 30 psi. Collision energy was normalized at 10%, 20%, 30%, and 40% HCD to study the different transitions of precursor ion to product ion and optimize collision energies for the MRM of selected molecules. Ultimately, a HCD of 10% was selected for the MRM transitions of *m/z* 365.1016 (pomiferin), 349.1068 (osajin), and 351.1226 (6,8‐diprenylgenistein). Standard solutions were prepared by performing 10‐fold serial dilutions of 17.4 mg/mL of **4**, 10.27 mg/mL of **7**, and 10.03 mg/mL of **8** in ACN containing 10% DMSO, with each dilution conducted in triplicate. Additionally, 15 g of the powdered fruits were soaked in 200 mL of EtOAc, mixed rigorously, heated at 50°C for 10 min in a water bath, filtered through a filter paper, and dried on a rotary evaporator. Of the 1.76 g of extract recovered, 4.43 mg were dissolved in 1 mL of 10% DMSO in ACN and serially diluted as above. A total of 20 calibration standards, along with the samples, were injected into the mass spectrometer with a resolution of 60 000 and a scan range of *m/z* 125–1800. Strong linearity was observed for all metabolites, with correlation coefficients exceeding 0.90. To improve sensitivity, accuracy, and specifically select parent ion and product ions corresponding to the mass of the molecules of interest, MRM was performed as the quantification tool. Data‐dependent acquisition (DDA) was conducted, where MS1 data were acquired in profile mode and MS^2^ data were acquired in centroid mode. Xcalibur software (version 4.2.47, Thermo Scientific, Waltham, MA, USA) was used for data acquisition and qualitative analysis, while the final quantitative analysis was performed using Skyline (version 24.1.0.199; Adams et al. [18]).

### 4.6 Antimicrobial Assays

MIC assays were used to determine the minimum concentration of an active ingredient required to inhibit half of the growth of microorganisms. Compounds were evaluated against a range of organisms, including the Gram‐positive bacterium, *S. aureus* ATCC6538, the Gram‐negative strain, *E. coli* ATCC8739, and the mould *Aspergillus brasiliensis* ATCC16404. Microbial solutions were prepared in saline (bacteria) and saline with tween (mould) and adjusted turbidometrically to a target concentration of 10^7^ ‐10^8^ CFU/mL. This inoculum solution was further diluted in Tryptone Soy broth (*S. aureus* and *E. coli*) or Soya Dextrose broth (*A. brasiliensis*) to achieve a final inoculum level of approximately 10^5^ CFU/mL for assay use. A stock 96‐well plate of the extracts was prepared in DMSO at a concentration of 20 mg/mL and serially diluted in DMSO. For each test plate, 5 *µ*L of each dilution (each well from the stock plate) was transferred to a new test plate, and 195 µL of inoculum in broth was added to each well. The *S. aureus* and *E. coli* plates were incubated on an orbital shaker for 18 ± 2 h at 32.5°C. The *A. brasiliensis* plates were inoculated at 20–25°C for 5 days. After incubation, the plates were visually assessed, and the MICs were determined as the most diluted well with reduced growth (∼50%) compared to the growth control. To determine the minimum biocidal concentration (MBC), 10 *µ*L from each well was pipetted onto neutralizing agar (Modified Letheen Agar with Tween) and incubated for 24 h at 32.5°C (*S. aureus* and *E. coli*) or 5 days at 20–25°C for 5 days (*A. brasiliensis)*. The MBC was determined as the most dilute concentration with no visible growth.

## Author Contributions

The manuscript was written through the contributions of all authors. All authors have given approval to the final version of the manuscript. **Gabin T.M. Bitchagno**: investigation, methodology, data curation, formal analysis, and writing – original draft; **Sohini S. Bhatia and Erin Garcia**: investigation, methodology, data curation, formal analysis, writing – review & editing, scott bintrim: project administration, writing – review & editing, supervision, and validation; **Paula Coates**: investigation, methodology, data curation, formal analysis, and writing – review & editing; **Debbie Mulligan and Scott Bintrim**: conceptualization, project administration, review & editing, supervision, and validation; **Monique S. J. Simmonds**: conceptualization, project administration, review & editing, supervision, and validation.

## Conflicts of Interest

The authors declare no conflicts of interest.

## Supporting information




**Supporting File 1**: cbdv70477‐sup‐0001‐SuppMat.docx

## Data Availability

The authors have nothing to report
