# Supplementary material for: Active Isoflavones of Osaje Orange Fruits against Staphylococcus aureus
Source: Chem Biodivers. 2025 Nov 8;22(12):e02220. doi: 10.1002/cbdv.202502220 (PMC12715979; doi:10.1002/cbdv.202502220)
Supplement: Supplementary file 1 — Supporting File 1: cbdv70477‐sup‐0001‐SuppMat.docx [file CBDV-22-e02220-s001.docx]

Active Isoflavones of Osaje Orange Fruits Against *Staphylococcus aureus*

Gabin Thierry M. Bitchagno^a^ *, Debbie Mulligan^b^, Paula Coates^c^, Erin Garcia^b^, Sohini Bhatia^b^, Scott Bintrim^b^, Monique S.J. Simmonds^a^ *

^a^ Royal Botanic Gardens, Kew, Richmond, London, TW9 3AE, g.bitchagnombahbou@kew.org and m.simmonds@kew.org

^b^ The Procter & Gamble Company, Mason Business Center, Mason, Ohio, USA

^c^The Procter & Gamble Company, 452 Basingstoke Rd, Reading, RG2 0RX, UK


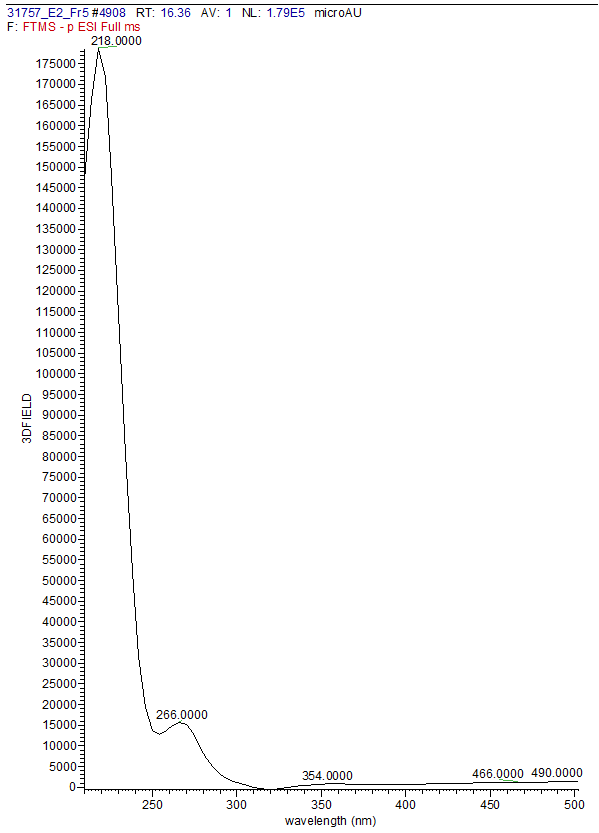


Figure S1. UV spectrum of compound **1**


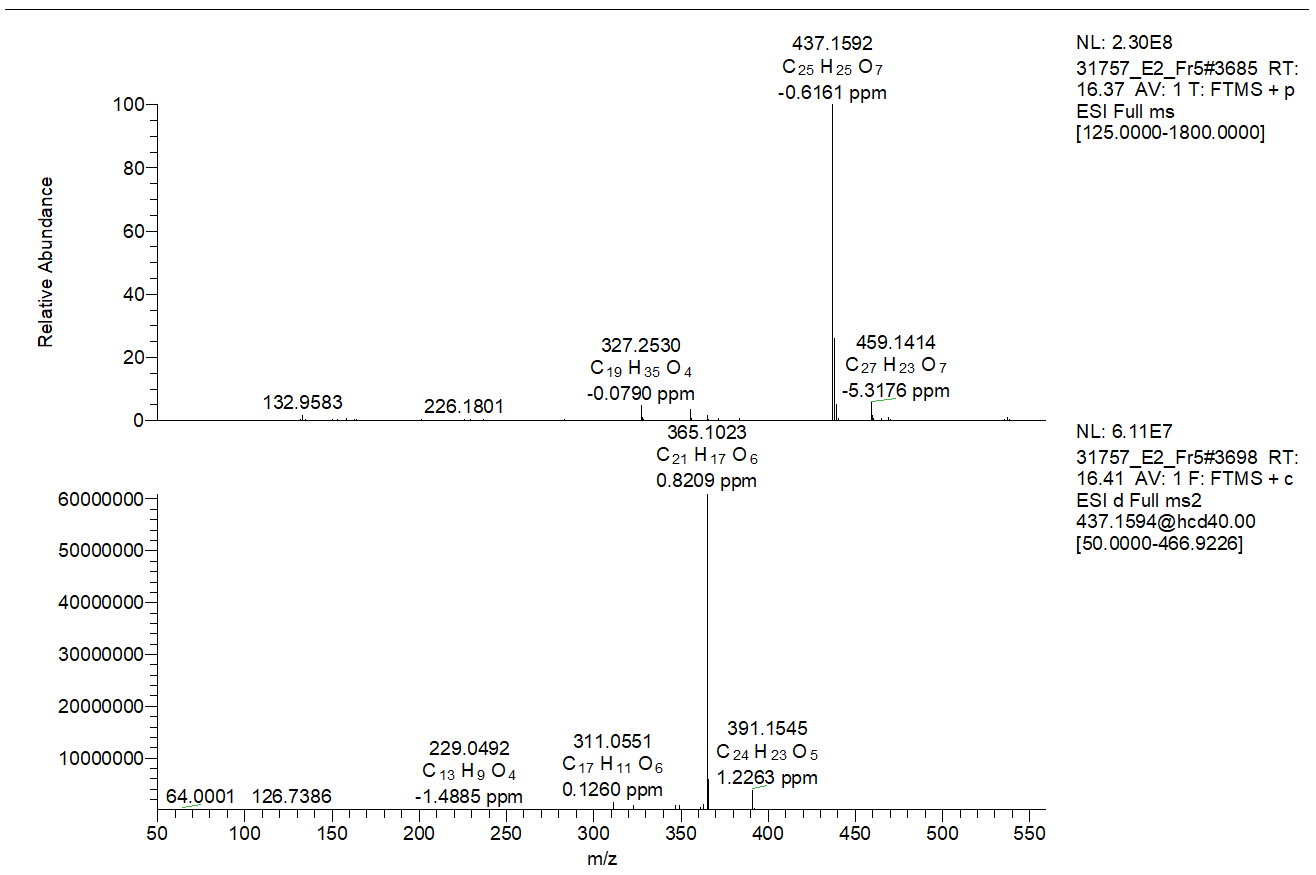


Figure S2. HRESI-MS/MS of compound **1**


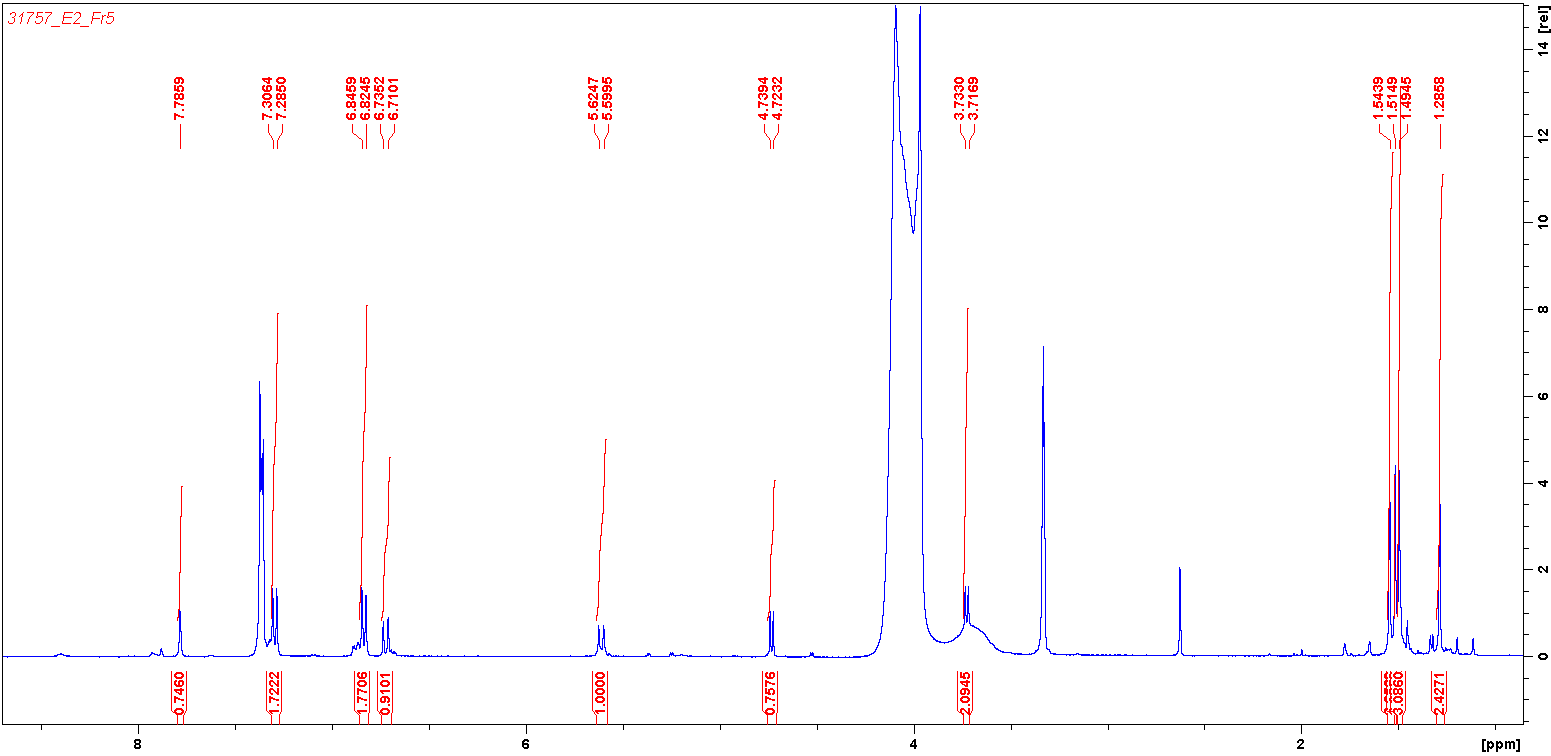


Figure S3. ^1^H NMR spectrum in MeOH-*d*_4_ of compound **1**


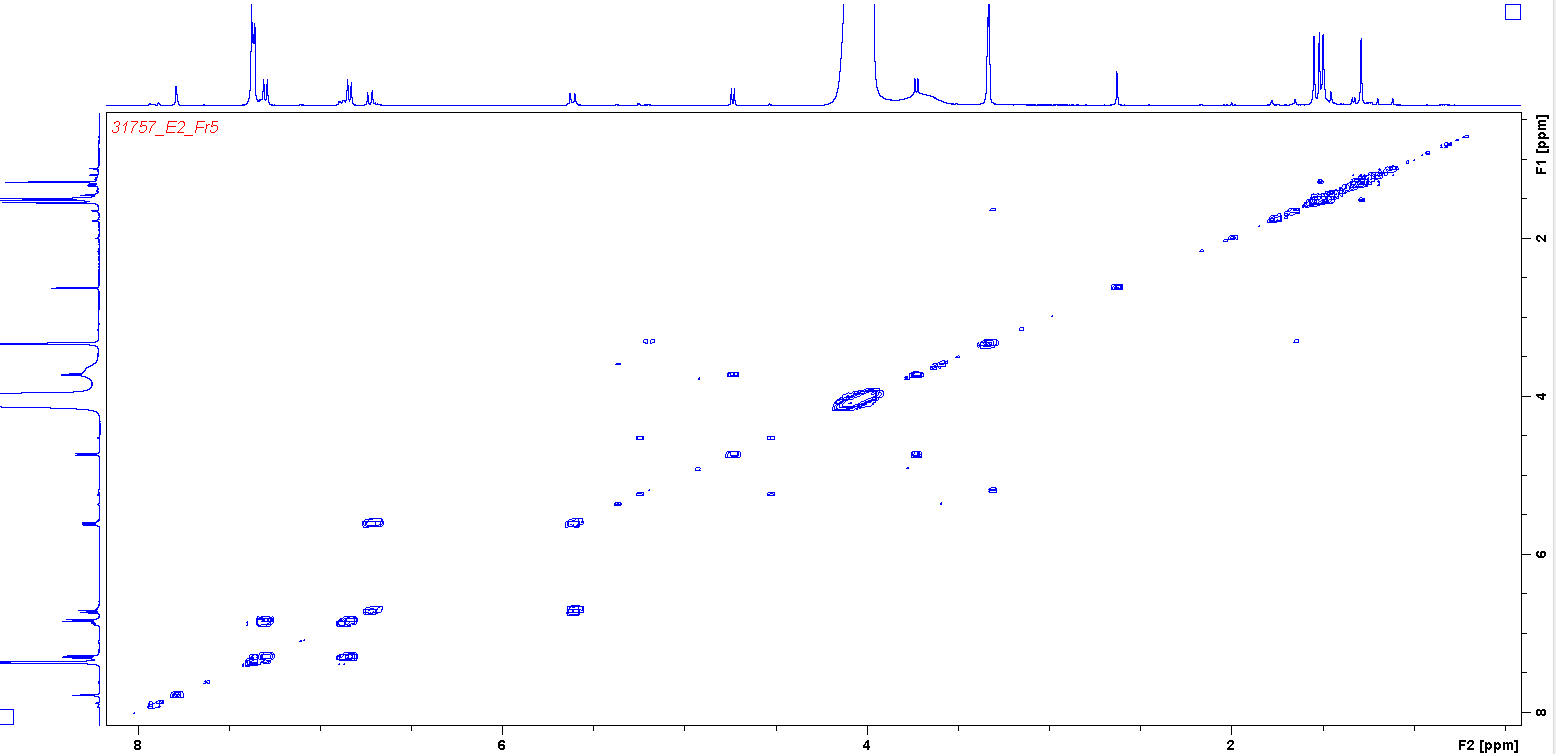


Figure S4. ^1^H, ^1^H COSY spectrum in MeOH-*d*_4_ of compound **1**


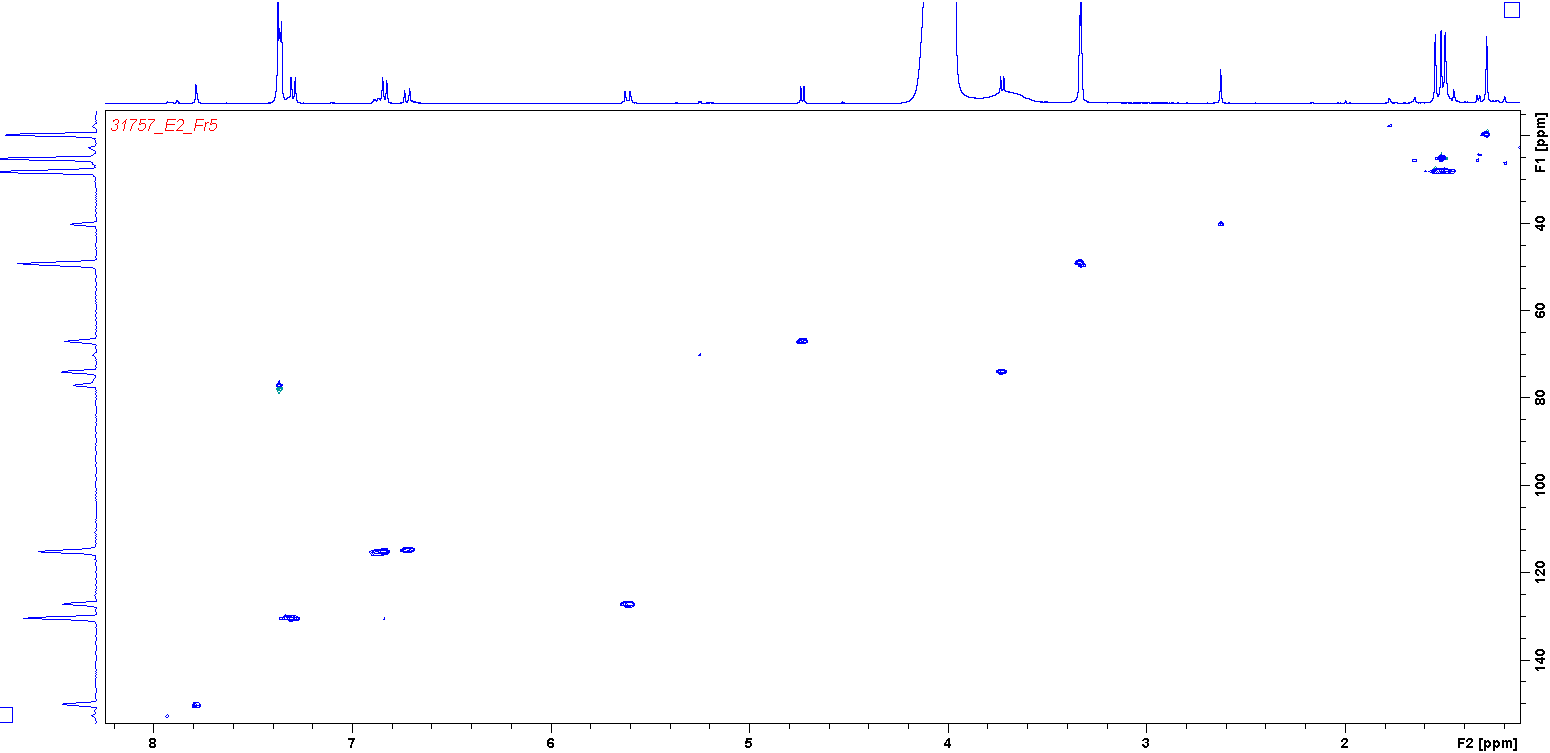


Figure S5. HSQC spectrum in MeOH-*d*_4_ of compound **1**


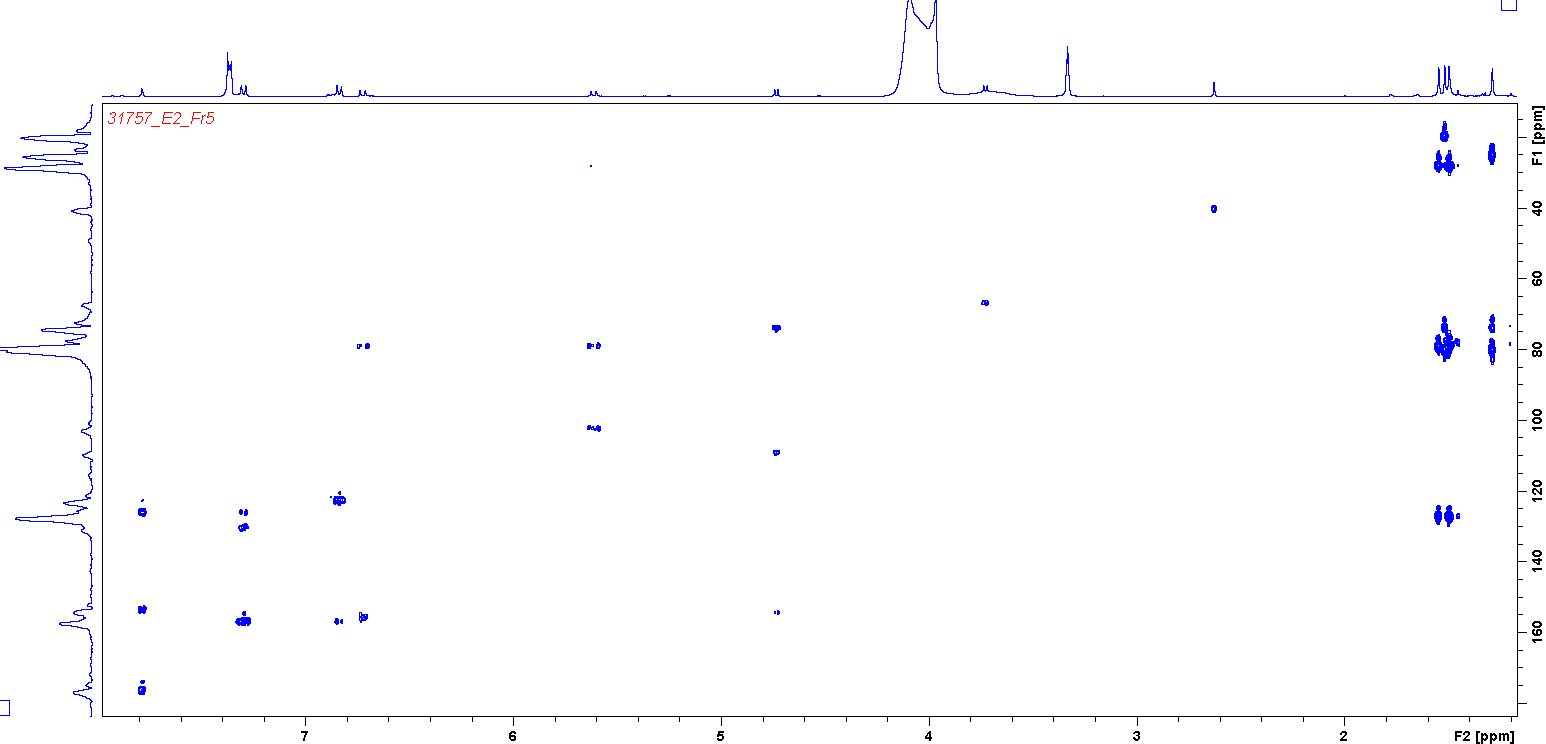


Figure S6. HMBC spectrum in MeOH-*d*_4_ of compound **1**


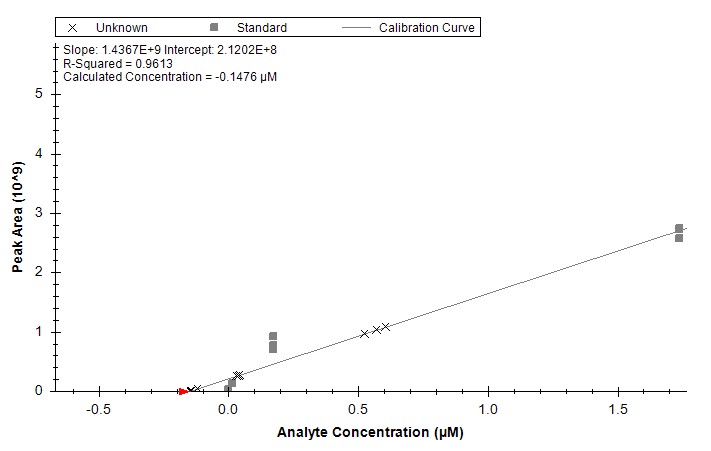


Figure S7. Calibration curve for the determination of diprenylgenistein (**4**)


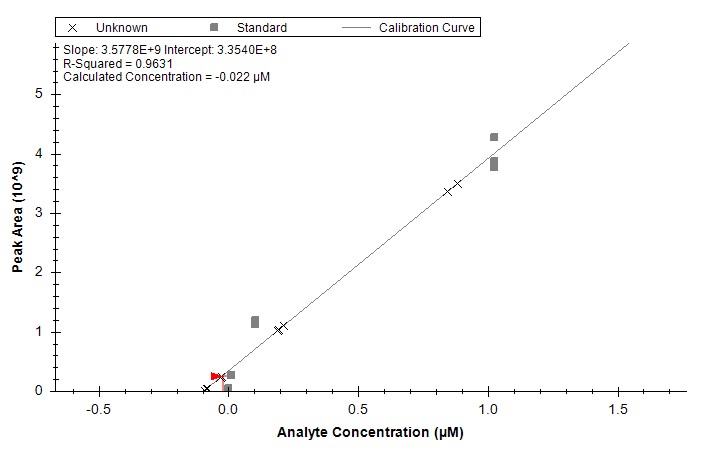


Figure S8. Calibration curve for the determination of osajin (**7**)


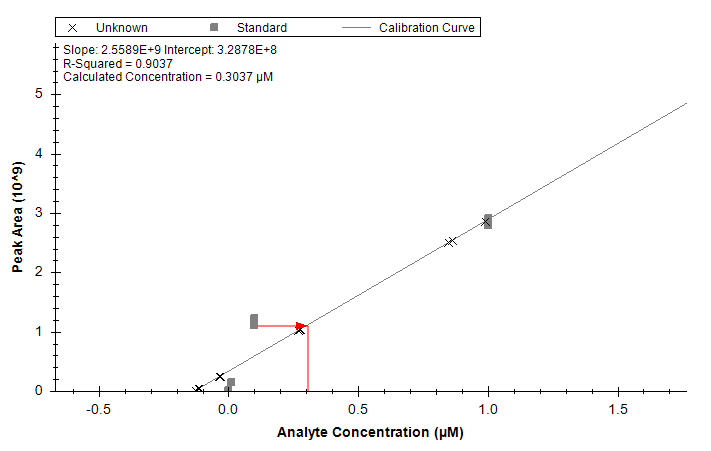


Figure S9. Calibration curve for the determination of pomiferin (**8**)
